# An Orbital Malignant Melanoma Arising in Cellular Blue Nevus in a Patient with Nevus of Ota

**DOI:** 10.7759/cureus.698

**Published:** 2016-07-18

**Authors:** Talayesa Buntinx-Krieg, Jie Ouyang, Mont Cartwright

**Affiliations:** 1 University of Central Florida College of Medicine; 2 Department of Pathology, Florida Hospital; 3 Medical Eye Associates, University of Central Florida College of Medicine

**Keywords:** nevus of ota, cellular blue nevus, malignant melanoma, melanoma, oculodermal melanosis, oculodermal melanocytosis, nevus fuscoceruleus ophthalmomaxillaris, congenital melanosis bulbi, orbit, orbital melanoma

## Abstract

Melanomas arising from orbital melanocytic proliferations are exceedingly rare. Many questions remain regarding their development and malignant transformation. We report on a 45-year-old Caucasian woman with a nevus of Ota that presented with visual disturbances involving her right eye and was found to have a biopsy-proven cellular blue nevus in the orbital space. Five years later, she presented with proptosis and worsening symptoms. Biopsy at that time showed a cellular blue nevus with areas of melanoma. We conclude that patients with a nevus of Ota or an orbital cellular blue nevus, particularly Caucasians, should be monitored for ocular/orbital involvement and followed closely for signs of rapid growth. There may be a progressive evolution to melanoma from a blue nevus.

## Introduction

Nevus of Ota presents with a bluish hyperpigmentation of the periocular region that may involve ocular and oral mucosal surfaces. The most common ophthalmic consequence is glaucoma, which occurs in 10% of patients with nevus of Ota [1]. There have been multiple reports of melanomas arising from the nevus of Ota involving the skin, ocular tissues, and the orbit [2]. We report a case in which a woman with a nevus of Ota developed a biopsy-proven orbital cellular blue nevus that underwent malignant transformation to malignant melanoma. 

## Case presentation

Informed patient consent was obtained for this patient's treatment. No identifying patient information is disclosed in this report.

**Figure 1:**
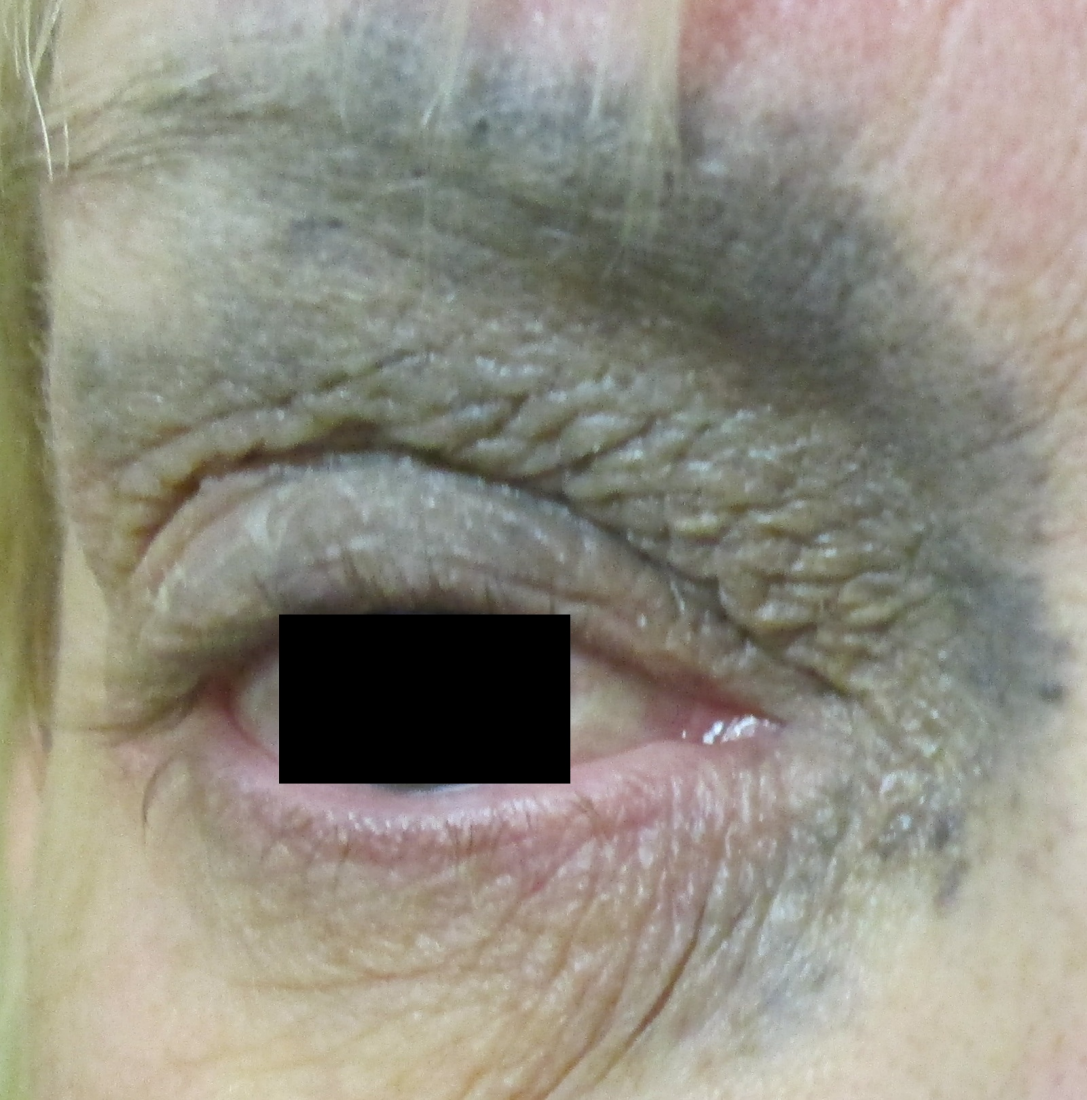
Nevus of Ota: Periorbital irregular blue patch as seen in our patient at presentation

A 45-year-old Caucasian woman with a diagnosis of right-sided nevus of Ota present since childhood presented with blurry vision, diplopia, photophobia, and pain localized to the right eye as well as signs of increased intracranial pressure (Figure 1).

**Figure 2:**
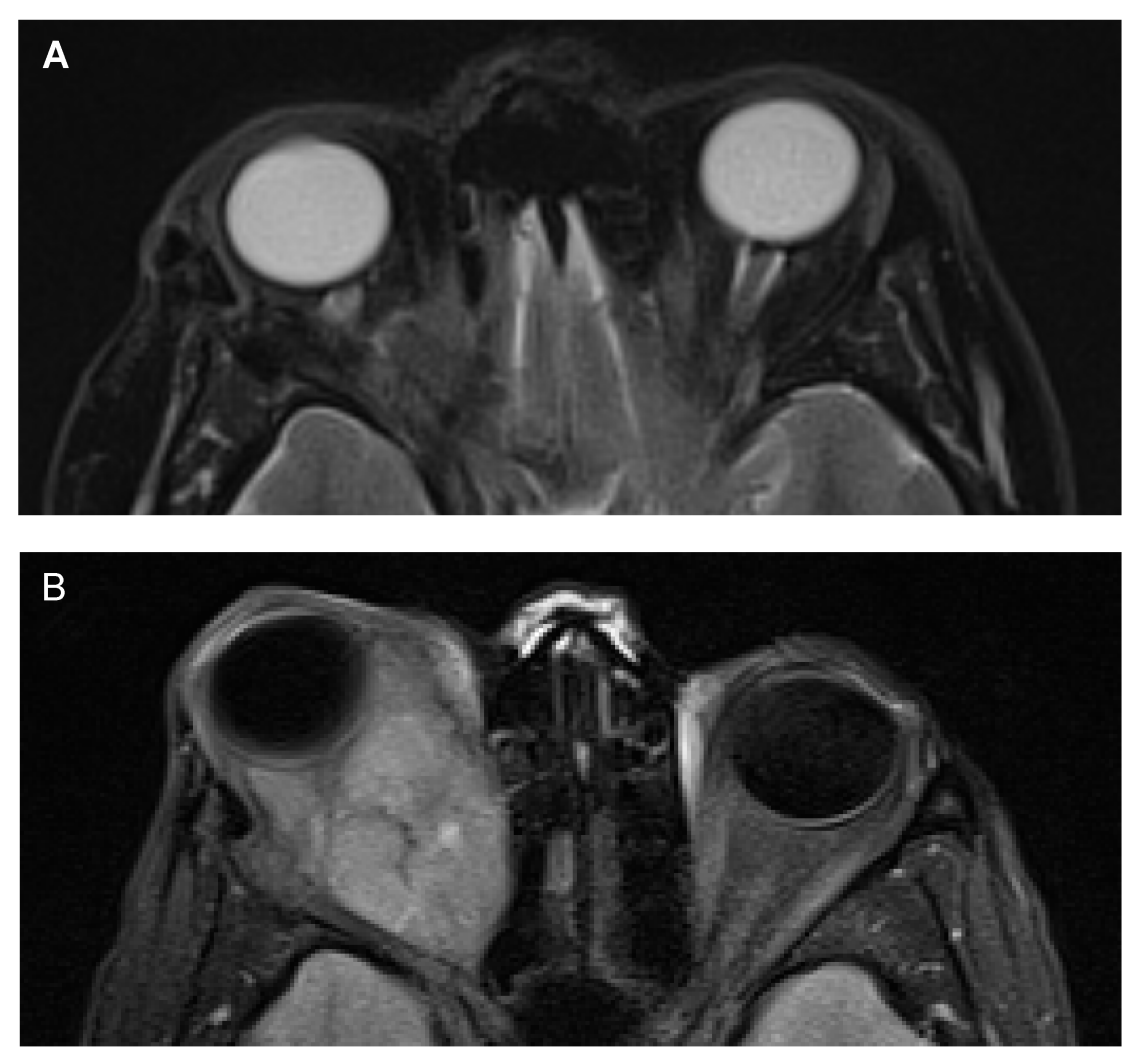
Orbital MRI A) The first post-surgical MRI showed a blue nevus remnant in the right orbit. B) Follow-up MRI approximately 5 years after initial presentation showed a lobulated posterior right orbital mass causing a significant proptosis that was pathologically described as a blue nevus with areas of high and low-grade melanoma.

Past medical history, family history, and social history were non-contributory. Her examination was significant for slight conjunctival injection, decreased extraocular eye movements, and evidence of ophthalmoplegia not indicative of any single cranial neuropathy in the right eye. The complete neurological exam was otherwise unremarkable. Computed tomography (CT) scan of the orbit revealed a non-enhancing right medial orbital apex mass measuring 1.7 x 1.7 x 1.6 cm. The orbital mass was displacing the optic nerve inferiorly and causing mass effect on the medial rectus muscle and right globe resulting in mild proptosis. During surgery, a diffuse infiltration of all orbital tissues with bluish-grey tissue was noted. Grossly, the tissue appeared tan-grey, soft, and friable, and the histopathologic diagnosis was a cellular blue nevus. Non-pigmented spindle cells in the biopsy immunostained positive for S-100 protein (a common marker for neural tissue/lesions and melanoma) and melanocytic specific markers, including human melanoma black-45 (HMB-45) protein and melanoma antigen recognized by T cells (MART-1). The MIB-1/Ki67 (a marker of cell proliferation) immunostain was positive in fewer than 3% of cells. At the one-week postoperative visit, there was a limitation in abduction, adduction, and infraduction, and visual acuity was 20/200 in the right eye and 20/20 in the left eye. Visual field testing showed a small inferior-nasal arcuate scotoma. Vision gradually improved in the right eye to 20/20 as did ocular motility. The first post-surgical magnetic resonance imaging (MRI) four months after presentation showed remnant blue nevus, and the patient was followed over the next three years with serial MRI scans that showed no changes (Figure 2A).

Three years after the initial presentation, there was a drop in visual acuity from 20/20 to 20/70 in the right eye, and shortly after this finding, she was lost to follow-up for eight months. At the next point of contact, she was found to have no light perception, a +3 pupillary afferent defect, increased retropulsion, exophthalmos with inferotemporal globe dystopia, and limited upward gaze in the right eye. She was given a tapered dose of steroids and pain medication. MRI scan one month later showed an orbital mass with cavitation and blood. It was assumed that the blue nevus had grown to a point where part of it had become necrotic. A decision to continue serial MRI monitoring was made, and four months later (approximately five years following initial presentation), imaging showed a large lobulated posterior orbital mass without cranial extension that showed significant changes from the previous MRI (Figure 2B).

Because of the profound proptosis and globe dystopia, the patient underwent a right anterior orbitotomy for debulking and removal of the growing mass. It could not be entirely removed due to its extensive growth in the orbital space, and patches of the pigmented tissue were left over the remainder of the orbit and soft tissues.

**Figure 3:**
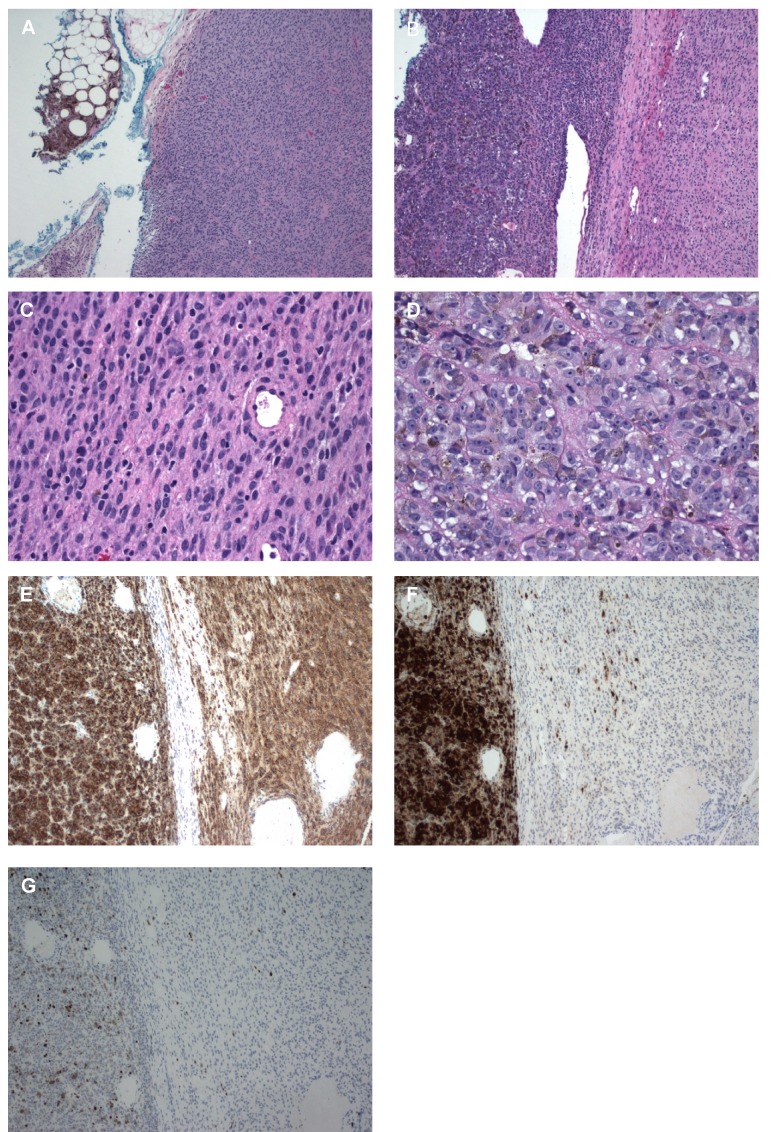
Histopathology and Immunohistochemistry of Orbital Mass A) (x10) The blue nevus component showing diagnostic pigmented spindle-shaped dendritic cells at left upper corner and adjacent amelanotic uniform oval to spindle cells with inconspicuous nucleoli and clear cytoplasm. B) (x10) Low power microscopic photo shows the benign blue nevus component (right) with an abrupt transition to distinctive malignant melanoma (left). C) (x40) Magnified benign component. D) (x40) Magnified malignant component shows pigmented melanocytes with prominent nucleoli, high nuclear to cytoplasmic ratio, and increased mitosis. E) (x10) Immunostain for HMB-45 shows both benign (right) and malignant (left) components that are strongly diffusely positive for HMB-45. F) (x10) Immunostain for p16 shows an abrupt distinction of benign (right) component with rare positive cells and a malignant component (left) with diffusely positive cells. G) (x10) Immunostain for MIB-1/Ki67 shows an abrupt distinction of benign (right) component with rare positive cells and malignant component (left) with a marked increase in proliferative index. A – D, hematoxylin and eosin.

Histopathological analysis at this time revealed two clear components. One was a low-grade blue nevus characterized by interweaving cells with increased cellularity and heavily pigmented spindle cells with a slight increase in mitotic cells; this component was similar to the previous biopsy. The other component was an epithelioid type melanoma with large hyperchromatic nuclei, prominent nucleoli, and cytoplasmic melanin (Figure 3). Immunohistochemical staining showed the tumor cells were strongly diffusely positive for HMB-45 and SOX10 (melanocytic specific markers). The melanoma cells showed diffuse strong cytoplasmic staining for p16 (a tumor suppressor protein) while scattered cells were positive for p16 in the blue nevus area. The tumor was negative for p53 (a tumor suppressor protein). MIB-1/Ki-67 (a marker of cell proliferation) immunostaining showed a proliferative index of 10% and 3% in the melanoma and blue nevus areas, respectively. The patient is going to be evaluated for metastases.

## Discussion

Nevus of Ota is defined histologically by the presence of dendritic melanocytes scattered between bundles of collagen in the upper third of the dermis without a sclerotic stromal reaction [3]. Contrastingly, cellular blue nevi, commonly found on the buttocks or scalp, are a typically benign variant of common blue nevi that are histologically characterized by dense cellularity and abundant melanin. The term “atypical cellular blue nevus” (ACBN) is used to describe borderline forms of cellular blue nevus with additional features (asymmetry, hypercellular foci, focal cytologic atypia, and occasional mitoses) that are suggestive of melanoma but not definitive [4]. There are few case reports describing the malignant potential of these histologically distinct lesions.

Melanoma arising in association with common or cellular blue nevi is very uncommon, and it is extremely peculiar for this to occur within the orbit. There are roughly 50 cases of nevus of Ota with cutaneous malignant transformation to melanoma. Melanomas of the orbit arising from isolated blue nevi or cellular blue nevi have been reported on at least four occasions [5-8]. Our case describes a patient with a nevus of Ota that developed an intraorbital cellular blue nevus with partial removal that ultimately underwent malignant transformation to an orbital melanoma, a specific sequence of events previously unreported in the literature.

Similar phenomena showing the malignant potential of nevus of Ota and cellular blue nevus have been reported. Medel, et al. described a Caucasian man with a nevus of Ota and ocular melanosis at the inferior fornix of the right eye that appeared to extend into the orbital space [4]. This patient had worsening proptosis, and a post-excisional diagnosis of atypical giant cellular blue nevus on a nevus of Ota was made. The lesion was removed in its entirety. Another case reported by Bisceglia, et al. described a Caucasian woman with a dermal cellular blue nevus with changes suspicious for malignant degeneration arising in a nevus of Ota [9]. Furthermore, Gerami, et al. reported a case of a nevus of Ota in a Caucasian woman showing progressive evolution to melanoma with intermediate states resembling cellular blue nevus [10]. These cases, taken in consideration with ours, support the notion that an ACBN may arise from a nevus of Ota within the orbit or skin and malignant transformation is a distinct possibility, particularly amongst Caucasians. However, it is important to note that the perceived Caucasian predominance may be due to a limited number of cases in the literature and overreporting of Caucasian cases over other races.

Epidemiologic studies describing malignant transformation rates in isolated cellular blue nevi or occurring in association with nevus of Ota are lacking due to the rare occurrence. It appears that these types of lesions behave more aggressively in Caucasians, as was the case in our patient. Given the limited information available, it is advisable to fully evaluate and biopsy patients presenting with new symptoms secondary to an orbital mass in association with a nevus of Ota. Histopathologic evidence of a cellular blue nevus or ACBN should raise concern for potential future malignancy.

## Conclusions

Patients with a nevus of Ota, particularly Caucasians, should be monitored for ocular/orbital involvement and followed closely for signs of rapid growth. Our case, in conjunction with the literature, suggests that there may be a progressive evolution to melanoma with stages including cellular blue nevus and ACBN.  
